# Comparison of changes in stress coping strategies between cognitive behavioral therapy and pharmacotherapy

**DOI:** 10.3389/fpsyt.2024.1343637

**Published:** 2024-04-15

**Authors:** Sakae Ihara, Nariko Katayama, Waka Nogami, Mizuki Amano, Sachiko Noda, Chika Kurata, Yuki Kobayashi, Yohei Sasaki, Dai Mitsuda, Mire Ozawa, Jun Matsuoka, Ryo Takemura, Hiroyuki Uchida, Atsuo Nakagawa

**Affiliations:** ^1^ Department of Neuropsychiatry, Keio University School of Medicine, Tokyo, Japan; ^2^ Department of Psychiatry, Tokyo-Adachi Hospital, Tokyo, Japan; ^3^ Department of Psychiatry, Higashi-Omiya Mental Health Clinic, Saitama, Japan; ^4^ Department of Psychiatry, Toyosato Hospital, Tsukuba, Japan; ^5^ Department of Psychiatry, Ogu Mental Health Clinic, Tokyo, Japan; ^6^ Clinical and Translational Research Center, Keio University Hospital, Tokyo, Japan; ^7^ Department of Neuropsychiatry, St. Marianna University School of Medicine, Kawasaki, Japan

**Keywords:** depression, cognitive behavioral therapy, task-oriented coping, distraction, social diversion

## Abstract

**Background:**

Coping refers to conscious responses to negative circumstances, with the intention of ameliorating these situations. Few studies have compared the differences between psychotherapy and medication treatment for coping strategies for depression. In this study, we investigated the differences in coping strategies between cognitive behavioral therapy (CBT) combined with medication (CBT group) and medication alone (pharmacotherapy group) among outpatients with depression.

**Methods:**

A prospective observational study was conducted among 50 patients with major depression (24 and 26 in the CBT and pharmacotherapy groups, respectively). Stress coping strategies (Coping Inventory for Stressful Situations [CISS]) and depression severity (Beck Depression Inventory-Second Edition [BDI-II]) were assessed at baseline and 16 weeks later. Changes in the CISS and BDI-II scores in both groups were tested using repeated analysis of variance. Inverse probability weighting with propensity score analysis was applied to address potential selection bias.

**Results:**

At 16 weeks, the CBT group exhibited increased CISS task-oriented coping, distraction, and social diversion scores, which differed from those of the pharmacotherapy group. The CBT group exhibited a significantly greater reduction in depressive symptoms than the pharmacotherapy group.

**Limitations:**

This study was not a randomized controlled trial and thus may have selection bias.

**Conclusion:**

Gaining adaptive coping skills, including task-oriented coping, distraction, and social diversion skills, by combining CBT with medication may lead to greater improvement in depression symptoms. These findings suggest that clinicians should evaluate coping strategies and facilitate the acquisition of adaptive coping strategies in patients with depression to reduce their symptoms.

## 1 Introduction

Major depression affects more than 280 million people worldwide (1), with a lifetime prevalence of approximately 6% in the Japanese population (2). Despite being a common mental disorder, the pathogenesis and causes of depression remain unclear. Among various hypothetical etiologies, the stress−diathesis model has been widely accepted, which states that major depression occurs due to the interaction between stressful events and individual characteristics such as personality, behavioral traits, and cognitive patterns (3). This model may explain how inadequate coping with stressful circumstances can lead to environmental non-adaptation and increase the risk of developing depression (4).

Coping is defined as responses to external stress and negative circumstances, with the intention of ameliorating these situations (5, 6). Endler and Parker (7) classified coping strategies into three basic dimensions: emotion-oriented coping, task-oriented coping, and avoidance-oriented coping. Emotion-oriented coping refers to person-oriented emotional responses, self-preoccupation, and fantasizing responses. Responses include lashing out at others or becoming angry at oneself, which aim to reduce stress. Task-oriented coping refers to purposeful task-oriented efforts to solve a problem by cognitively restructuring the problem or attempting to alter the circumstances; the emphasis is on the task or planning and attempts to solve the problem. Avoidance-oriented coping refers to activities and cognitive changes aimed at avoiding stressful circumstances by distracting oneself with other tasks or via social diversion as a means of alleviating stress. Based on an empirical study, Endler and Parker (7) reported that avoidance-oriented coping could be further divided into two subdimensions as follows: distraction and social diversion. The distraction subdimension involves substituting activities to avoid stressful situations (task-oriented avoidance), whereas the social diversion subdimension involves seeking connections and communication with others (person-oriented avoidance).

Improving coping strategies serves as a protective factor that decreases the adverse effects of stressors and reduces depressive symptoms. Several studies have revealed that higher scores on task-oriented coping and social diversion were associated with milder depressive symptoms, whereas higher scores on emotion-oriented coping were associated with more significant depressive symptoms (8, 9). It has also been observed that the same coping strategy can have different effects in different contexts. For example, shopping can worsen depressive symptoms in university students preparing for exams, while improving depressive symptoms in chronically depressed patients (9). Furthermore, a literature review of longitudinal studies of mood disorders demonstrated that reduced use of emotion-oriented coping and increased use of task-oriented coping predict increased remission and decreased risk of relapse (4). In a prior clinical study, task-oriented coping increased after 16 weeks of antidepressant use (10). Another study showed that inappropriate emotion-oriented coping decreased after 8 weeks of selective serotonin reuptake inhibitor use (11). Enhancing the acquisition of adaptive coping skills through psychotherapy alleviates emotional distress (12).

Cognitive-behavioral therapy (CBT) is a psychotherapy modality that aims to help individuals develop coping strategies that facilitate problem-solving by challenging and realistically modifying maladaptive cognitions and behaviors which negatively affect patients’ mood and behavior (13, 14). For example, maladaptive automatic thoughts such as excessive self-blame can cause resulting behaviors such as spending excessive time in bed; however, if the validity of such automatic thoughts is tested and the patient realizes that they were overly generalized, these negative feelings are relieved and functional behaviors can be developed (14). Several studies have reported that adaptive coping increases with CBT. Serran et al. (15) reported that task-oriented coping and social diversion increased in offenders after 4 months of group CBT. Moreover, the enduring effects of CBT have been associated with gaining adaptive coping strategies (16). Therefore, to validate the role of these adaptations in the treatment process, we focused on the changes in coping strategies which occur before and after CBT.

Although several studies have indicated that coping strategies for depression change with treatment, few studies have compared the differences between psychotherapy and pharmacotherapy. Therefore, we compared differences in the changes in coping strategies caused by CBT combined with medication (CBT group) and medication alone (pharmacotherapy group) among outpatients with depression. We hypothesized that adaptive coping strategies, including task-oriented coping and social diversion, would increase among those who received CBT more than those who received medication alone.

## 2 Methods

### 2.1 Patients

This was a prospective observational study in which patients were recruited between November 2020 and January 2023. Patients were considered eligible for the study if they met the following inclusion criteria: 1) diagnosis of major depressive disorder according to the Diagnostic and Statistical Manual of Mental Disorders, 4th Edition (DSM-IV); and 2) age between 20 and 75 years at study enrollment. The main exclusion criteria were as follows: 1) serious or unstable physical illness; 2) unstable psychiatric symptoms, including an imminent risk of self-harm or harm; and 3) study participation was deemed inappropriate by the attending physician.

The study psychiatrists clinically assessed the patients during the baseline interviews. The study was conducted at four medical facilities, including one university hospital, two psychiatric hospitals, and one mental health clinic. Informed consent was obtained from each patient before enrollment in the study. This study was approved by the Research Ethics Committee of Keio University School of Medicine (approval no. 20200088) and was conducted in accordance with the tenets of the Declaration of Helsinki.

Patients in the CBT group were provided face-to-face CBT sessions of approximately 50 min scheduled weekly for 16 weeks after baseline (16 weeks), with up to four additional CBT sessions allowed if deemed necessary by the therapist. The therapists followed the procedures described in the CBT Therapist Manual for Depression-Japanese Ministry of Health, Labour and Welfare (17). This manual is based on Beck’s original manual with slight modifications to accommodate the cultural characteristics of Japanese patients. The program consisted of the following six components. 1) psychoeducation and assessment; 2) case conceptualization, problem clarification, and behavioral activation; 3) identification of mood and automatic thoughts; 4) validation of automatic thoughts and problem solving; 5) identification of beliefs; and 6) summary and relapse prevention. At the beginning of every session, the patient and therapist cooperatively determined the agenda, which was discussed during the session. “Homework” was then assigned at the end of the session and the patient practiced these skills between sessions. The CBT therapists comprised two psychiatrists and six clinical psychologists with master’s or doctoral degrees. All therapists received CBT training, which included a 2-day intensive workshop and 1-h onsite group supervision every week from a skilled CBT supervisor (AN), with thorough reviews of the cases and feedback to maintain adherence to the CBT Therapist Manual. All patients received pharmacotherapy with their psychiatrist alongside CBT.

Patients in the pharmacotherapy group were treated with medication by two psychiatrists for a mean length of 14.5 (standard deviation [SD]: 0.7) years. In addition to pharmacological management, the patients’ side effects were reviewed, and supportive guidance was provided during the medication visit. No restrictions were imposed on medication management.

### 2.2 Procedures

Interviews for the diagnosis of major depressive disorder were conducted using the DSM-IV Structured Clinical Interview. Baseline was defined as the time of study enrollment, at which time, all patients underwent a semi-structured interview with a study psychiatrist and psychologist. In addition, patients were interviewed to collect demographic data and information on clinical characteristics, including age, sex, years of education, illness duration, and current medications. Patients completed self-report questionnaires, including the Coping Inventory for Stressful Situations (CISS) and Beck Depression Inventory-Second Edition (BDI-II), at baseline and 16 weeks.

### 2.3 Measures

#### 2.3.1 CISS

The CISS is a self-reported questionnaire developed by Endler and Parker (7) to assess multidimensional coping in stressful situations. We used the Japanese version of the CISS, which has been confirmed to be both reliable and valid (18). The CISS consists of three primary categories as follows: task-oriented coping (16 items), avoidance-oriented coping (16 items), and emotion-oriented coping (16 items). In addition, avoidance-oriented coping consists of the following two subcomponents: distraction (eight items) and social diversion (five items). Respondents indicated whether they engaged in various coping activities during stressful situations on a five-point scale (1 = not at all to 5 = very much).

The CISS task-oriented coping refers to problem-solving orientation and the drafting and implementation of problem-solving strategies and is consistent with the basic principles of problem-solving (19). The items include “use the situation to prove my ability,” “analyze the problem before reacting,” “come up with several different solutions to the problem,” and “determine a course of action and follow it”.

As for the CISS avoidance-oriented coping subcomponent, distraction refers to becoming absorbed in specific tasks to avoid stressful situations (7). It includes items such as “window shop,” “treat myself to my favorite food or snack,” and “go out for a snack or meal.” Another CISS avoidance-oriented coping subcomponent was social diversion, which refers to being with others to avoid stressful situations (7). It includes items such as “try to be with other people,” “visit a friend,” and “phone a friend”.

The CISS emotion-oriented coping refers to excessive emotional responses, such as lashing out at people or getting angry at oneself. These responses may increase stress and are considered nonadaptive coping (7); the items include “blaming myself for procrastination,” “becoming very tense,” and “taking it out on others”.

#### 2.3.2 BDI-II

The BDI-II is a self-report questionnaire developed by Beck (20) to measure depression severity in adults and adolescents. Symptom severity was rated based on a prior 2-week timeframe and evaluated on a four-point scale (0–3), with higher scores indicating greater depression severity. We used the Japanese version of the BDI-II, and its reliability and validity have been previously confirmed (21).

#### 2.3.3 Defined daily dose of antidepressants at baseline

The defined daily dose (DDD) is the unit of measurement of daily dosage recommended by the World Health Organization (22). One DDD is the assumed average daily maintenance volume of a drug used in adults as the primary indication. In this study, only antidepressants were included; if more than one antidepressant was administered, the values for each antidepressant were combined.

### 2.4 Statistical analyses

Chi-square tests were used for categorical variables, and unpaired *t*-tests were used for continuous variables to compare demographic data, clinical characteristics, and depression severity (BDI-II) between the CBT and pharmacotherapy groups.

Inverse probability weighting with propensity score (IPW) analysis was used to address the potential selection bias for the treatment. Adjustments were made using IPW to homogenize the two patient groups, to account for any differences in background factors. The following factors were included as covariates in the IPW analysis: sex, work status, marital status, cohabitation, family history of psychiatric disorders, history of suicide attempts, age, total years of education, total number of lifetime depressive episodes, median lifetime duration of depressive episodes, median duration of current depressive episodes, depression severity at baseline (BDI-II), and DDD (22) of antidepressants and antipsychotic drug use at baseline. Weights were calculated using the calculated propensity scores, and IPW analysis was performed.

IPW is an extension of the propensity score method and is commonly used in observational studies to enhance causal inference. IPW has the advantage of including all samples, without reducing the sample sizes (23). Therefore, we adopted this method for the analysis in this study.

Group differences in mean baseline and endpoint (16 weeks) changes over time as mean scores on the primary scale and subcomponents of the CISS were tested using repeated analysis of variance (ANOVA). Items that were significantly different between the two groups, even after adjusting for IPW, were included in the model as covariates. Time (baseline or 16 weeks) was used as the “within-subjects” variable, and group (CBT or pharmacotherapy group) was used as the “between-groups” variable. The data underwent a false discovery rate (FDR) correction to control for multiple comparisons (no more than 5% false positives on average) (24). We performed two-sided t-tests to compare CISS scores at baseline and at 16 weeks between groups. All data underwent false discovery rate (FDR) correction to control for multiple comparisons for the five measures in each group (false positives averaged less than 5%) (24).

Between-group differences at baseline and 16 weeks, as mean scores of BDI-II and DDD, were analyzed similarly for the CISS. All statistical analyses were performed using SPSS Statistics for Windows (version 29.0).

## 3 Results

### 3.1 Patient demographic and clinical characteristics

The demographic and clinical characteristics at baseline and the comparison between the CBT and pharmacotherapy groups are presented in **Table 1**. Fifty patients participated in the study: 24 in the CBT group and 26 in the pharmacotherapy group. Approximately half of the patients in this study sample were women (*n* = 24, 48%); most were young-to-middle-aged (mean age: 39.4 years, SD, 12.5). The duration of current depressive episodes was more than 1 year (mean: 14.8 months, SD, 18.0), and most were moderately depressed (mean BDI-II score: 26.4, SD, 9.7). Unadjusted, the CBT group had a significantly larger proportion of women (*p* = 0.049), more family history of psychiatric disorders (*p* = 0.04), and more total years of education (*p* = 0.01). The DDD scores of antidepressants tended to be greater in the CBT group. However, the DDD scores of antipsychotic drugs were not significantly different between groups. There were no significant differences between the two groups in the median lifetime duration of depressive episodes or median duration of current depressive episodes. After adjusting for IPW, the two groups became similar except for the total years of education (*p* = 0.03).

**Table 1:** Comparison of demographic and clinical characteristics between CBT and Pharmacotherapy group.

Demographic and clinical characteristics	CBT	pharmacotherapy		CBT	pharmacotherapy	
	(n=24)	(n=26)				
	Unadjusted			After adjusting by IPW		
	n (%)	n (%)	*p*-value^a^	n (%)	n (%)	*p*-value^a^
**Female, n (%)**	15 (62.5)	9 (34.6)	0.049*	24 (60.0)	28 (53.8)	0.56
**Working status**			0.94			0.63
Employed, housewife, or student	9 (37.5)	10 (38.5)		16 (40.0)	23 (45.1)	
Others (unemployed and on sick leave)	15 (62.5)	16 (61.5)		24 (60.0)	28 (54.9)	
**Marital status**			0.94			0.37
Married	9 (37.5)	10 (38.5)		14 (35.0)	23 (44.2)	
Single, divorced, or widowed	15 (62.5)	16 (61.5)		26 (65.0)	29 (55.8)	
**Cohabiting**	8 (33.3)	8 (30.8)	0.85	12 (29.3)	11 (21.6)	0.40
**Family history of psychiatric disorders**	11 (45.8)	5 (19.2)	0.04*	17 (42.5)	19 (36.5)	0.56
**Past history of suicide attempts**	1 (4.2)	3 (11.5)	0.34	1 (2.5)	3 (5.9)	0.44
	Mean (SD)	Mean (SD)	*p*-value^b^	Mean (SD)	Mean (SD)	*p*-value^b^
**Age (years)**	36.8 (12.6)	41.9 (12.1)	0.15	38.8 (11.7)	42.9 (12.5)	0.11
**Total years of education**	15.3 (2.2)	13.4 (2.6)	0.01*	14.7 (2.4)	13.7 (2.3)	0.03*
**Total number of lifetime depressive episodes**	1.4 (0.8)	1.6 (0.9)	0.49	1.4 (0.7)	1.4 (0.7)	0.97
**Median lifetime duration of depressive episodes (months)**	67.3 (101.3)	46.9 (66.3)	0.40	54.4 (92.3)	51.8 (61.8)	0.87
**Median duration of current depressive episodes (months)**	19.7 (22.6)	10.3 (10.9)	0.07	16.0 (19.5)	16.0 (13.5)	0.99
Depressive severity at baseline						
BDI-II	28.0 (10.3)	24.9 (9.0)	0.27	27.8 (10.1)	27.0 (8.5)	0.68
Medication dose						
DDD (Antidepressant)	1.04 (0.74)	0.68 (0.72)	0.09	0.98 (0.79)	1.02 (0.88)	0.80
DDD (Antipsychotic drugs)	0.05 (0.12)	0.04 (0.10)	0.69	0.06 (0.12)	0.09 (0.14)	0.24

^a^p-value is for unpaired t-test. ^b^p-value is for Chi-squared test. *p< 0.05.

CBT, cognitive behavioral therapy; SD, standard deviation; IPW, inverse probability weighting; BDI-II, Beck Depression Inventory-II; DDD, defined daily dose.

### 3.2 Treatment engagement

The treatment engagements are presented in **Table 2**. All patients in the CBT group completed the weekly CBT course.

**Table 2:** Differences in treatment engagement between CBT and Pharmacotherapy group.

Variable	CBT (n=24)	pharmacotherapy (n=26)	
	Mean (SD)	Mean (SD)	*p*-value
**Number of medication visits**	14.7 (3.5)	9.3 (3.9)	< 0.001*
**Number of CBT sessions attended**	14.5 (1.5)	N/A	N/A

p-values are for unpaired t-test. *p < 0.05.

CBT, cognitive-behavioral therapy; SD, standard deviation; N/A, not applicable.

### 3.3 Comparison of changes in CISS scores between baseline and 16 weeks

The changes in the mean scores on the primary components and subcomponents of the CISS for both groups using repeated-measures ANOVA are listed in **Table 3**. The total years of education significantly differed between the groups, even after adjusting for IPW; therefore, repeated-measures ANOVA was conducted with this as a covariate.

**Table 3:** Differences in CISS alteration between CBT and Pharmacotherapy group (Analysis after IPW adjustment).

	CBT			pharmacotherapy			Group by time interaction F	*p*-value^b^
	Baseline	16 weeks	*p*-value^a^	Beseline	16 weeks	*p*-value^a^		
	Mean (SD)	Mean (SD)		Mean (SD)	Mean (SD)			
CISS								
** Task**	44.2 (10.6)	50.0 (11.3)	0.014*	37.4 (9.7)	37.8 (10.5)	0.712	(1,73) = 9.34	0.003*
** Avoidance**	43.7 (6.4)	49.5 (8.3)	< 0.001*	43.4 (10.2)	41.9 (10.6)	0.224	(1,86) = 17.38	< 0.001*
** **Distraction	44.8 (6.9)	48.2 (8.8)	0.009*	45.9 (9.6)	42.4 (9.7)	0.068	(1,86) = 10.80	0.001*
** **Social diversion	41.9 (8.2)	49.3 (11.1)	< 0.001*	40.8 (11.5)	40.4 (10.4)	0.796	(1,86) = 11.66	< 0.001*
** Emotion**	60.7 (8.6)	54.4 (13.5)	< 0.001*	65.6 (13.0)	52.4 (10.9)	< 0.001*	(1,86) = 3.87	0.053

p-value^a^ is for paired t-test. p-value^b^ is for Repeated-measure ANOVA. *FDR-corrected p < 0.05

CISS, Coping Inventory for Stressful Situations; CBT, cognitive behavioral therapy; IPW, Inverse Probability Weighting; ANOVA, analysis of variance; SD, standard deviation; Task, Task-oriented coping; Avoidance, Avoidance-oriented coping; Emotion, Emotion-oriented coping.

The CBT group had increased scores for CISS task-oriented coping, CISS avoidance-oriented coping, CISS distraction, and CISS social diversion after 16 weeks (*p* = 0.014, *p* < 0.001, *p* = 0.009, and *p* < 0.001, respectively). Furthermore, the scores differed between the two groups (F (1, 73) = 9.34, *p* = 0.003; F (1, 86) = 17.38, *p* < 0.001; F (1, 86) =10.80, *p* =0.001; and F (1, 86) = 11.66, *p* < 0.001).

### 3.4 Other clinical assessments

Changes in the mean BDI-II and DDD scores for the two groups using repeated-measures ANOVA are presented in **Table 4**. The total years of education significantly differed between the groups, even after adjusting for IPW; therefore, repeated-measures ANOVA was conducted with this as a covariate. The BDI-II scores decreased more significantly in the CBT group than in the pharmacotherapy group (F (1, 85) = 15.76, *p* < 0.001). The DDD score increased in the pharmacotherapy group after 16 weeks (*p* < 0.001).

**Table 4:** Differences in BDI-II and DDD alteration between CBT and Pharmacotherapy group (Analysis after IPW adjustment).

	CBT			Pharmacotherapy			Group by time interaction F	*p*-value^b^
	Baseline	16 weeks	*p*-value^a^	Baseline	16 weeks	*p*-value^a^		
	Mean (SD)	Mean (SD)		Mean (SD)	Mean (SD)			
Depressive severity								
BDI-II	27.8 (10.1)	14.1 (10.7)	< 0.001*	27.0 (8.5)	20.7 (14.4)	< 0.001*	(1,85) = 15.76	< 0.001*
Medication dose								
DDD (Antidepressant)	0.98 (0.79)	1.01 (0.85)	0.707	1.02 (0.88)	1.41 (0.97)	< 0.001*	(1,85) = 4.84	0.031*
DDD (Antipsychotic drug)	0.06 (0.11)	0.07 (0.10)	0.348	0.09 (0.14)	0.10 (0.15)	0.348	(1,85) = 0.002	0.968

p-value^a^ is for paired t-test. p-value^b^ is for Repeated-measure ANOVA.

BDI-II, Beck Depression Inventory-II; DDD, Defined daily dose; CBT, cognitive behavioral therapy; IPW, Inverse Probability Weighting; ANOVA, analysis of variance.

## 4 Discussion

In this study, we compared the differences in the changes in coping strategies between CBT combined with pharmacotherapy and pharmacological treatment alone among outpatients with depression. We observed that adaptive coping strategies increased in those who received CBT, which was confirmed by improved scores on the CISS task-oriented coping, social diversion, and distraction tests. In addition, we demonstrated that depressive symptoms were alleviated for those who received CBT alongside pharmacological treatment compared with those who received pharmacotherapy alone.

The increase in CISS task-oriented coping scores in the CBT group suggests that CBT improves task-oriented coping acquisition. CISS task-oriented coping refers to processes of problem-solving that coincide with the steps of problem-solving techniques in Beck’s CBT Manual for Depression (7, 13). In Beck’s CBT Manual for Depression, problem-solving techniques were addressed in the following steps: 1) problem-solving orientation; 2) clarifying the problem and defining the goals and outcomes; 3) establishing an action plan; 4) selecting the best action plan from those identified; 5) enacting the best action plan; and 6) evaluating the degree of success of the outcome (25). Furthermore, CBT for depression is a problem-solving-oriented approach in which the patient and therapist cooperatively solve the patient’s problems (25, 26). A study comparing patients with depression and healthy individuals showed that patients with depression were more negatively problem-oriented and less capable of generating problem-solving strategies (27). In a study of 80 college students, Nezu and Ronan (28) reported that those with depressive symptoms were less able to determine the best effective solutions. Problem-solving techniques reduce depressive symptoms by mitigating deficiencies such as negative problem-solving orientation, lower ability to generate solutions, and lower ability to determine effective problem solutions (29, 30). Notably, in a large meta-analysis that investigated which internet CBT components were most efficacious for depression, suggestive evidence showed that problem-solving was one of the most beneficial components (iMD: −0.64; 95% confidence interval: −1.41, 0.09) (31). Therefore, the increase in CISS task-oriented coping after CBT indicates an acceleration in the problem-solving process.

In our sample, the CISS social diversion score increased after CBT. This finding is in agreement with the evidence from a randomized controlled trial of group CBT, which showed an association between increased communication with others and alleviation of depressive symptoms among depressed university students (16). Social diversion is a coping strategy that involves connecting with others to manage stress (7). The questionnaire items included in the CISS social diversion, such as “try to be with other people” and “visit a friend,” coincided with action plans when working out with behavioral activation sessions. Beck (13) posits that behavioral activation promotes engagement in reinforcing personal and meaningful activities (communicating with others) that lead to a positive mood. Thoits (32) asserted that connection with others improves depression by obtaining behavioral guidance and purpose and meaning in life. Notably, all patients in our study had 50-min face-to-face sessions for 16 weeks, and all engaged in behavioral activation during therapy. Taken together, our findings of increased social diversion coping after CBT indicated that the patients sought more advice and informational support and received more sympathy and understanding.

In this study, the CISS distraction scores increased in the CBT group. Distraction is a task-oriented avoidance strategy that involves shifting focus to another task to manage stressful situations (7). This finding is in line with a study showing that distraction was negatively related to concurrent depressive symptoms (*r* = −0.24) and significantly predicted decreases in depressive symptoms over time (*pr* = −0.27) (33). Although some reports have described distraction as a maladaptive coping strategy that promotes poor outcomes (34), other evidence suggests that distraction is adaptive. Distraction predicts improved life satisfaction and self-efficacy (35). Another study reported that “positive” distraction was related to increased well-being and positive emotions and fewer depressive symptoms when controlling for avoidance among those under a chronic stressor (35, 36). Indeed, the median lifetime duration of depressive episodes of patients in our sample was nearly 5 years, indicating that almost all of them were under a chronic stressor.

### 4.1 Limitations

This study has several limitations that warrant discussion. First, this study did not use a randomized controlled design but an IPW to address the selection bias inherent in the prospective observational design. Furthermore, there is a possibility of unmeasured confounders. Second, although pharmacotherapy was provided by an experienced psychiatrist, it was not controlled. Third, there were only two evaluation points: baseline and 16 weeks. It is possible that the effects of CBT may emerge later. Longer follow-up evaluations may provide more information. Finally, only the BDI-II, a self-reported depression scale, was used to assess depression severity. The assessment would be more robust if observer-rated scales were used. These limitations warrant more relevant future studies.

### 4.2 Conclusions

In conclusion, combining CBT with pharmacotherapy can increase task-oriented coping, distraction, and social diversion skills. Gaining these adaptive coping strategies through therapy may result in greater improvement of depression symptoms.

These findings suggest that clinicians should evaluate the coping strategies of patients with depression and provide interventions that promote the acquisition of adaptive coping strategies, which may lead to successful treatment of depression. In the future, conducting randomized controlled trials to further test these results and clarify the association between changes in stress coping and improvements in depression is warranted.

## Data availability statement

The raw data supporting the conclusions of this article will be made available by the authors, without undue reservation.

## Ethics statement

The studies involving humans were approved by the Research Ethics Committee of Keio University School of Medicine (approval no. 20200088). The studies were conducted in accordance with the local legislation and institutional requirements. The participants provided their written informed consent to participate in this study.

## Author contributions

SI: Conceptualization, Data curation, Formal analysis, Project administration, Resources, Visualization, Writing – original draft, Writing – review & editing. NK: Conceptualization, Data curation, Formal analysis, Project administration, Resources, Visualization, Writing – review & editing. WN: Investigation, Methodology, Writing – review & editing. MA: Investigation, Methodology, Writing – review & editing. SN: Investigation, Methodology, Writing – review & editing. CK: Investigation, Methodology, Writing – review & editing. YK: Investigation, Methodology, Writing – review & editing. YS: Investigation, Methodology, Writing – review & editing. DM: Investigation, Methodology, Writing – review & editing. MO: Investigation, Methodology, Writing – review & editing. JM: Formal analysis, Methodology, Resources, Writing – review & editing. RT: Formal analysis, Methodology, Writing – review & editing. HU: Project administration, Resources, Writing – review & editing. AN: Conceptualization, Formal analysis, Project administration, Resources, Visualization, Writing – review & editing.
